# Iron deficiency and iron supplementation in heart failure: a dynamic phenotype and a moving therapeutic target

**DOI:** 10.1093/eschf/xvag057

**Published:** 2026-02-17

**Authors:** Jan Biegus

**Affiliations:** Department of Cardiology, Clinical Department of Intensive Cardiac Care, Faculty of Medicine, Wroclaw Medical University, Institute of Heart Diseases, Borowska 213, Wroclaw 50-556, Poland; Department of Cardiology, Jan Mikulicz Radecki University Hospital in Wrocław, Wroclaw 50-556, Poland

**Keywords:** Iron deficiency, Definition, Congestion, Therapy

Iron deficiency (ID) is highly prevalent in patients with heart failure (HF) and has consistently been associated with impaired exercise capacity, reduced quality of life, and worse clinical outcomes, independent of and extending beyond traditional measures of disease severity.^1–5^ Iron is integral to multiple pathophysiological relevant processes (like: cellular energy metabolism, mitochondrial function, oxygen transport, inflammation, and immune regulation) and its correction represents a therapeutic target across several mechanistic theories proposed to explain both HF pathogenesis and its clinical manifestations.^6–8^ Moreover, the bidirectional interaction between ID and HF creates self-perpetuating mechanisms that drive disease progression, worsens myocardial function and clinical outcomes.^4,9–11^ As a consequence, contemporary HF guidelines recommend routine screening for ID and support intravenous iron supplementation—most notably ferric carboxymaltose—in iron-deficient HF patients.^12–14^ Despite more than a decade of randomized controlled trials (RCTs), iron repletion in HF has not convincingly demonstrated a robust reduction in mortality, leaving a significant gap between biological plausibility, symptomatic benefit, and hard clinical endpoints.^15^

Meta-analyses of available RCTs have shown a consistent and clinically meaningful reduction in combined endpoint (cardiovascular death and total cardiovascular hospitalizations) and in HF hospitalizations with intravenous iron therapy, whereas effects on cardiovascular or all-cause mortality typically fall just short of statistical significance.^16,17^

This pattern is once again confirmed in the recent meta-analysis published in ESC Heart Failure, which synthesizes evidence from over 6400 patients enrolled in 11 RCTs of ferric carboxymaltose in HF.^18^ In that analysis, intravenous iron significantly reduced recurrent HF hospitalizations (by ∼25–31%) and the composite of hospitalization or cardiovascular death, while mortality outcomes showed only a non-significant trend towards benefit, particularly attenuated with longer follow-up. Iron supplementation also improved the 6-min walk distance by ∼29 m. A consistent pattern seen across endpoints is the time-dependent reduction in the effects of iron supplementation, seen regardless of the clinical endpoint analysed, with longer follow-up periods linked to smaller effect sizes.

## Iron supplementation should not be viewed as a one-time intervention

In line with the meta-analysis’s conclusions, the effects of iron therapy on long-term clinical outcomes should be interpreted with caution and remain a subject for future research. It is biologically and clinically plausible that the impact of a single intervention attenuates over time, particularly as not all RCTs incorporated a predefined re-dosing strategy during follow-up. Importantly, iron supplementation does not address the underlying mechanisms that lead to ID in HF; it simply replenishes depleted iron stores. Consequently, the identification of ID should also be regarded also as the identification of patients at increased risk of future iron deficiency, including recurrence after initial correction.

These findings, together with the totality of available evidence, raise at least two fundamental, still unresolved questions.

## Is the current definition of iron deficiency in HF optimal?

Perhaps the most critical issue is the definition of ID itself.^5^ The current guideline-endorsed definition—based on ferritin <100 µg/L or ferritin 100–299 µg/L with transferrin saturation (TSAT) <20%—was adopted largely on pragmatic grounds rather than through outcome-driven optimization. Ferritin, an acute-phase reactant, is profoundly influenced by inflammation, congestion, and hepatic dysfunction—hallmarks of HF—raising concern that this definition may misclassify biologically relevant ID in a substantial proportion of patients.

Emerging evidence increasingly suggests that TSAT-based definitions may better reflect functional iron deficiency and identify patients who derive the greatest clinical benefit from iron supplementation. Importantly, cohorts with lower TSAT appear to have a higher risk profile and may represent a subgroup in which iron repletion could plausibly reduce hospitalizations and improve survival. Thus, the repeated ‘near-miss’ of mortality significance observed across trials and meta-analyses may reflect suboptimal patient selection rather than a true absence of biological effect.

## Why does iron supplementation reduce HF hospitalizations?

A second intriguing question concerns the mechanism underlying the consistent reduction in recurrent HF hospitalizations by iron repletion. Iron plays a central role in mitochondrial energy production, skeletal and myocardial muscle function, and potentially renal tubular energetics—all processes fundamental to exercise tolerance, congestion handling, and volume homeostasis. Theoretically, though, correction of ID might improve functional reserve, improve energetics, enhance diuretic responsiveness, and stabilize vulnerable patients during periods of physiological stress.

Given that congestion is a key pathophysiological driver of most episodes of decompensation and HF hospitalizations, one may reasonably speculate that achieving a meaningful reduction in the annual risk of HF hospitalization—estimated at approximately 30%—would require an intervention that directly improves congestion-related pathways. Despite these plausible mechanisms, a direct causal pathway linking iron repletion to reduced risk of decompensation in HF has not been definitively established. Moreover, most trials were not designed to explore mechanistic endpoints and dose–response relationships. If, however, the pathway of reducing the risk of HF hospitalization through modulation of congestion (fluid and electrolyte homeostasis) is not the primary mechanism, this raises the question of what direct mechanism underlies the observed reduction with iron supplementation.

## Where do we go from here?

The accumulating evidence leaves little doubt that intravenous iron improves symptoms and reduces the risk of HF hospitalizations. The remaining challenge is not whether iron therapy works, but for whom, how, and based on which definition of ID it works best. Future studies should move beyond the current one-size-fits-all definition and focus on outcome-oriented phenotyping of ID, potentially anchored in TSAT-driven or other biomarker-guided strategies.

## Declarations

### Data Availability

No new data were generated or analysed in support of this editorial. All data discussed are derived from previously published studies.
